# A first description of the Colombian national registry for rare diseases

**DOI:** 10.1186/s13104-017-2840-1

**Published:** 2017-10-26

**Authors:** Heidi Eliana Mateus, Ana María Pérez, Martha Lucía Mesa, Germán Escobar, Jubby Marcela Gálvez, José Ivo Montaño, Martha Lucía Ospina, Paul Laissue

**Affiliations:** 1 Center For Research in Genetics and Genomics-CIGGUR, GENIUROS Research Group, School of Medicine and Health Sciences, Universidad del Rosario, Carrera 24 No. 63C-69, Bogotá, Colombia; 2 Ministerio de Salud y Protección Social, Bogotá, Colombia; 3 Instituto Nacional de Salud, Bogotá, Colombia

**Keywords:** Rare disease registry, Orphan disease, Colombian health system

## Abstract

**Objective:**

Orphan diseases must be considered a public health concern, underlying country-specific challenges for their accurate and opportune diagnosis, classification and management. Orphan disease registries have not yet been created in South America, a continent having a population of ~ 415 million inhabitants. In Colombia ~ 3 million of patients are affected by rare diseases. The aim of the present study was to establish the first Colombian national registry for rare diseases. The registry was created after the establishment of laws promoting the development of clinical guidelines for diagnosis, management, census and registry of patients suffering rare diseases.

**Results:**

In total, 13,215 patients were recorded in the Colombian registry. The survey reported 653 rare diseases. The most common diseases were congenital factor VIII deficiency (hemophilia A) (8.5%), myasthenia gravis (6.4%), von Willebrand disease (5.9%), short stature due to growth hormone qualitative anomaly (4.2%), bronchopulmonary dysplasia (3.9%) and cystic fibrosis (3.2%). Although, a marked under-reporting of cases was observed, some pathologies displayed similar behavior to that reported by other initiatives and databases. The data currently available in the registry provides a baseline for improvement regarding local and regional surveys and the start for better understanding rare diseases in Colombia.

**Electronic supplementary material:**

The online version of this article (10.1186/s13104-017-2840-1) contains supplementary material, which is available to authorized users.

## Introduction

Orphan diseases represent a highly heterogeneous group of disorders which can affect any organ. Such pathologies involve a wide range of clinical features and severity. To date, 5000–8000 rare diseases have been recognized and most of them have a genetic origin [1, 2]. Rare diseases in the USA have been defined as those affecting less than 1 in 1250 individuals (1/2000 in the European Union) [1, 3, 4]. It has been estimated that 6–8% of the total population is affected by rare diseases, representing ~ 30 and ~ 25 million people in the European Union and the USA, respectively [5].

Rare disease patient registries are essential for collecting clinical and epidemiological data thereby leading to better understanding of their pathogenesis as well as contributing towards drug development and effective therapeutic responses. Various national registries for rare diseases have been created (especially in North America and Europe) which could provide the basis for expanded international initiatives leading to more comprehensive and homogeneous data being collected [6, 7]. For instance, countries having covering systematic collections of data regarding specific rare diseases (or a group of diseases) would include France, Switzerland, Italy, Spain, the United Kingdom, Germany, the Netherlands, Norway, Sweden, Estonia and Poland [7, 8]. Further well-developed databases and initiatives for identifying patients affected by rare diseases include the Orphanet for rare diseases and orphan drugs, the Rare Disease-HUB (RD-HUB), the National Organization for Rare Disorders (NORD), the NIH/NCATS GRDR program and the Rare Diseases Clinical Research Network (RDCRN) [2, 9–11].

Orphan disease registries have not yet been created in South America, a continent covering 14 countries and having an estimated population of 415 million inhabitants. Some associations and databases including specific pathologies have been created in Colombia and South American countries as the Colombian registry for Pompe disease and the LATAM fabry registry [12].

However as yet, no structures for the systematic recording of rare diseases have been established. The present study provides a first description of a Colombian national registry for rare diseases. The main objective of the registry was to collect epidemiological data from patients affected by rare diseases and to propose distinct future national initiatives. These include the creation of diagnosis/treatment guidelines and the construction specialized medical centers for rare diseases.

## Main text

### Methods

#### Establishing a national registry

Detailed information on the steps for establishing the registry has been included as Additional files 1 and 2.

### Results

**Fig. 1:**
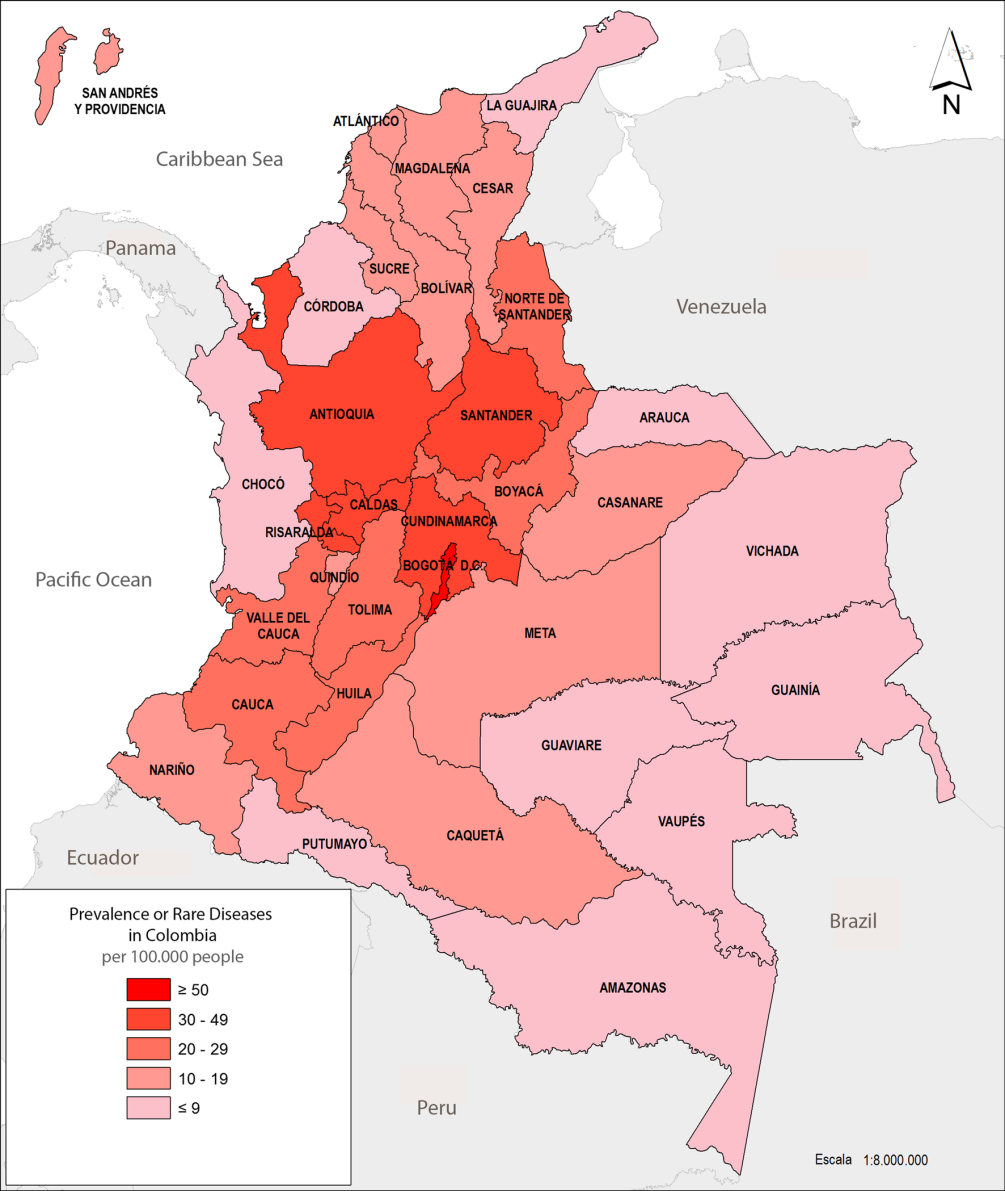
Prevalence of rare diseases in Colombia. Data corresponds to all living patients affected by rare diseases registered in the health care provider’s system (2015)

In total, 13,215 patients were recorded in the Colombian registry: 54% (n = 7132) female and 46% (n = 6083) male. Population characteristics are summarized in Tables 1 and 2. Their mean age was 32 years old (SD 23.3; CI 95% 31.61–32.39) and 1199 (9.1%) individuals suffered disability on enrollment. The largest numbers of patients were enrolled in Bogotá (32.65%) and the Antioquia region (14.33%). The Vaupés and Amazonas departments had the lowest percentage of patients reported in the registry (0.01 and 0.02%, respectively). Figure 1 represents prevalence by region (Colombia is a Unitarian Republic, which is divided administratively and politically into 33 regions: 32 departments and Bogotá).

**Table 1:** Most common disorders reordered in the Colombian registry for rare diseases

Disease	N	%	Prevalence per 100.000	n (Female)	Prevalence per 100.000	n (Male)	Prevalence per 100.000
Factor VIII deficiency	1117	8.5	2.37	77	0.32	1.040	4.47
Myasthenia gravis	839	6.4	1.78	570	2.39	269	1.16
Von Willebrand disease	779	5.9	1.65	551	2.31	228	0.98
Short stature due to growth hormone qualitative anomaly	559	4.2	1.19	203	0.85	356	1.53
Bronchopulmonary dysplasia	511	3.9	1.08	239	1.00	272	1.17
Cystic fibrosis	424	3.2	0.90	209	0.88	215	0.92
Diffuse cutaneous systemic sclerosis	408	3.1	0.87	331	1.39	77	0.33
Guillain–Barré syndrome	392	3.0	0.83	169	0.71	223	0.96
Idiopathic and/or familial pulmonary arterial hypertension	377	2.9	0.80	248	1.04	129	0.55
Acquired von Willebrand syndrome	281	2.1	0.60	211	0.88		0.00
Factor IX deficiency	270	2.0	0.57	73	0.31	197	0.85
Marfan syndrome	234	1.8	0.50	101	0.42	133	0.57
Acromegaly	232	1.8	0.49	134	0.56	98	0.42
Primary biliary cirrhosis	208	1.6	0.44	187	0.78	21	0.09
Multiple sclerosis-ichthyosis—factor VIII deficiency	196	1.5	0.42	147	0.62	49	0.21

Data corresponds to all living patients affected by rare diseases registered in the health care provider’s system (2015)

**Table 2:** Distribution of rare diseases by nosologic group (ICD-10)

ICD-10	n	%	Female	Female%	Male	Male%	0–18 years	%	> 18 years	%
I	9	0.1	4	0.1	5	0.1	4	0.1	5	0.1
II	146	1.1	87	1.2	59	1.0	72	1.3	74	1
III	3455	26.1	1461	20.5	1994	32.8	1357	25	2098	26.9
IV	2040	15.4	1046	14.7	994	16.3	1222	22.5	818	10.5
V	128	1.0	66	0.9	62	1.0	52	1	76	1
VI	2120	16.0	1191	16.7	929	15.3	375	6.9	1745	22.4
VII	149	1.1	75	1.1	74	1.2	48	0.9	101	1.3
VIII	39	0.3	21	0.3	18	0.3	8	0.1	31	0.4
IX	478	3.6	298	4.2	180	3.0	92	1.7	386	5
X	15	0.1	8	0.1	7	0.1	8	0.1	7	0.1
XI	457	3.5	378	5.3	79	1.3	25	0.5	432	5.5
XII	512	3.9	292	4.1	220	3.6	120	2.2	392	5
XIII	987	7.5	792	11.1	195	3.2	122	2.2	865	11.1
XIV	32	0.2	20	0.3	12	0.2	17	0.3	15	0.2
XVI	546	4.1	261	3.7	285	4.7	536	9.9	10	0.1
XVII	2092	15.8	1130	15.8	962	15.8	1364	25.1	728	9.4
XVIII	3	0.0	2	0.0	1	0.0	3	0.1	0	0
XIX	7	0.1	0	0.0	7	0.1	4	0.1	3	0
Total	13,215	100.0	7132	100	6083	100	5429	41.1%	7786	58.9%

Data corresponds to all living patients affected by rare diseases registered in the health care provider’s system (2015)

International Statistical Classification of Diseases and Related Health Problems (ICD-10): Chapter I (certain infectious and parasitic diseases), Chapter II (neoplasms), Chapter III (diseases of the blood and blood-forming organs and certain disorders involving the immune mechanism), Chapter IV (endocrine, nutritional and metabolic diseases), Chapter V (mental and behavioural disorders), Chapter VI (diseases of the nervous system), Chapter VII (diseases of the eye and adnexa), Chapter VIII (diseases of the ear and mastoid process), Chapter IX (diseases of the circulatory system), Chapter X (diseases of the respiratory system), Chapter XI (diseases of the digestive system), Chapter XII (diseases of the skin and subcutaneous tissue), Chapter XIII (diseases of the musculoskeletal system and connective tissue), Chapter XIV (diseases of the genitourinary system), Chapter XV (pregnancy, childbirth and the puerperium), Chapter XVI (certain conditions originating in the perinatal period), Chapter XVII (congenital malformations, deformations and chromosomal abnormalities), Chapter XVIII (symptoms, signs and abnormal clinical and laboratory findings, not elsewhere classified), Chapter XIX (injury, poisoning and certain other consequences of external causes), Chapter XX (external causes of morbidity and mortality), Chapter XXI (factors influencing health status and contact with health services), Chapter XXII (codes for special purposes)

The survey reported 653 rare diseases (~ 33% of all diseases included in the Colombian list of rare diseases). Sixty-nine percent of these had a genetic origin. The most common diseases were congenital factor VIII deficiency (hemophilia A) (8.5%), myasthenia gravis (6.4%), Von Willebrand disease (5.9%), short stature due to growth hormone qualitative anomaly (4.2%), bronchopulmonary dysplasia (3.9%) and cystic fibrosis (3.2%). The top 20 most common pathologies were identified in ~ 50% of reported cases. The most common occurring pathologies in males were factor VIII deficiency (4.5%), short stature due to growth hormone qualitative anomaly (1.2%), bronchopulmonary dysplasia (3.9%), myasthenia gravis (1.2%) and Von Willebrand disease (1%). The top five rare diseases most commonly occurring in females were myasthenia gravis (8%), Von Willebrand disease (7.7%), diffuse cutaneous systemic sclerosis (4.7%), idiopathic and/or familial pulmonary arterial hypertension (3.5%) and bronchopulmonary dysplasia (3.4%) (Table 1). According to the International Statistical Classification of Diseases and Related Health Problems (ICD-10), the most prevalent pathologies were diseases of the blood and blood forming organs (Chapter III) (26.1%), endocrine, nutritional and metabolic diseases (Chapter IV) (16%), congenital malformations, deformations and chromosomal abnormalities (Chapter XVII) (15.8%), nutritional and metabolic diseases (15.4%) and diseases of the musculoskeletal system and connective tissues (Chapter XIII) (7.5%) (Table 2). The most commonly occurring diseases amongst patients aged under 18 years old were congenital malformations, deformations and chromosomal abnormalities (25.1%), diseases of the blood and blood forming organs (25%), endocrine nutritional and metabolic diseases (22.5%), certain conditions originated in the perinatal period (9.9%) and diseases of the eyes and adnexa (6.9%). The most commonly occurring pathologies in adults were diseases of the blood and blood forming organs (27%), diseases of the eye and adnexa (22.4%), diseases of the musculoskeletal system and connective tissue (11.1%), endocrine, nutritional and metabolic diseases (10.5%) and congenital malformations, deformations and chromosomal abnormalities (9.4%) (Table 2).

### Discussion

It has been estimated that the prevalence of rare diseases is between 6–8% [13]. This value was estimated taking into account 5000–8000 disease while less than 2000 were included in the present version of the registry. Thus, a marked under-reporting of cases was observed in our study, since rare disease prevalence was 28/100,000 inhabitants (0.028%). This scenario highlights the fact that > 2.5 million patients have not been recorded or have not been diagnosed. Some of the most common diseases recorded in the registry’s present version (e.g. hemophilia A and myasthenia gravis) have been reported as having high prevalence in other databases (Orphanet) (Table 1). It has been reported that factor VIII deficiency prevalence ranges between 12.8 ± 6 patients per 100.000 in high income countries while in other parts of the world it has been estimated at 6.6 ± 4.8 [14]. The present study showed that 1117 individuals were affected by factor VIII deficiency, having 4.5/100,000 prevalence similar to that (4.6) reported by the World Federation of Hemophilia for Colombia (2014) [15]. However, preliminary predictions had estimated that 5000 individuals should be affected by this pathology in Colombia [16]. A preliminary national survey has already reported a higher number of cases (n = 1497) which pinpoints mistakes during their present recording. Indeed, a very rare disease, multiple sclerosis-ichthyosis-factor VIII deficiency (< 20 cases having been described worldwide to date) was reported as affecting 196 individuals in the present version of the registry [17]. This scenario suggests that these patients were probably affected by factor VIII deficiency alone.

As previously stated, regarding countries having limited resources, low hemophilia prevalence values in the present version of the registry might have been related to its inaccurate diagnosis and the unavailability of pertinent treatment thereby leading to premature death [18]. The above, added to shortcomings related to inappropriate record-keeping procedures, might be the main variables for its under-registry.

Myasthenia gravis, a chronicle debilitating autoimmune muscular disorder, has 1.5–17.9 prevalence worldwide (7.7–37.7 in Europe) [19]. A first study of myasthenia gravis in Colombia’s Antioquia region revealed a 2.7 prevalence while the present study reported 1.78 cases per 100,000 habitants [20], suggesting doctors having good skills regarding its diagnosis.

Interestingly, our group has found that, similarly to the report by Mazzucato et al. [21], some disorders displayed high prevalence during adulthood as haematological, musculoskeletal, eye and congenital diseases [21]. This might have been due to the fact that these pathologies, which frequently lead to disability (especially working limitations), are relatively well-diagnosed and treated. In a recent report, a novel strategy based on diagnostic codes, allowed the construction of a rare disease registry for patients from Western Australia [22]. This study was particularly interesting as it permitted to estimate the impact of rare diseases on the costs of discharges.

Most neoplasias (representing around 5% of all of rare diseases in other countries) were also excluded from the present version of the Colombian registry because they are covered by specific legislation [23]. In other registries neoplastic diseases prevalence ranges from 3.6 to 14.5% [21, 22]. It is worth stressing that several infectious diseases (e.g. tuberculosis, leishmaniasis) which are considered as rare in other countries, due to their low prevalence, frequently occur in Colombia since they have greater than 1/5000 prevalence and were thus excluded from the registry. Interestingly, for some groups of diseases, we have found differences between males and females. For instance, diseases of the blood and blood forming organs (Chapter III) affected 20.5 and 32.8% of women and males, respectively. This might have been due to the fact that common blood disorders (e.g. haemophilia A and B) are X-linked recessive diseases predominantly affecting males. In the contrary, females are more commonly affected than males (11.1% vs 3.2%) by diseases of the musculoskeletal system and connective tissue (Chapter XIII) which includes rheumatoid arthritis and polyarthritis (Table 2).

Our group has observed that the Colombian regions most affected by under-registry (e.g. the islands of San Andrés and Providencia and the Arauca and Chocó departments) have traditionally been related with poverty. This data suggested that information regarding rare diseases across those Colombia’s vulnerable areas was due to population barriers to health access (Fig. 1). Although 5000–8000 (5833 in the Orphanet Database) rare diseases have been defined (1920 of which have been considered by Colombian legislation), our survey only included 653 of them. This might have been due to the fact that many rare diseases involve multi-system organ damage/failure; this might lead to their inaccurate classification by doctors who have not been trained in identifying such kinds of disorder. Furthermore, ICD-10, which was taken as the basis for classifying disorders in the present version of our registry, lacks numerous rare diseases (Table 2). Health systems have recently been recommended to use ORPHA codes, apart from ICD-10, for classifying rare diseases to improve the above [24]. Future ICD-11 is planned for use in 2018, promises an expanded repertoire of rare diseases which might improve the accuracy of identifying/classifying many disorders. It should be noted that misdiagnosis frequently occurs in Colombia due to some rare diseases’ inherent complexity regarding their clinical and molecular diagnosis, together with few medical geneticists being available (about 60 all around the country). Such drawbacks surely merit accurate epidemiological follow-up and study regarding challenging, rare diseases. Underreporting is expected to be resolved in near future, at least in part, as it has been proposed that doctors should report rare diseases directly.

The data currently available in the registry, which is the first attempt to systematically record patients suffering rare diseases in Colombia, provides a baseline for improvement regarding local and regional surveys. This first approach for rare diseases registration will be continued through a program called SIVIGILA (National Vigilance System of Public Health) created as a mandatory tool for specialists and expertise centers to report patients with orphan diseases. This information must be the start for better understanding rare diseases in Colombia. It is clear that the data summarized in the present report has to be interpreted with caution as the reader must be aware that it has several limitations, especially due to many diseases having been inaccurately classified.

The on-going and systematic recording of data by well-trained physicians should ensure the high quality (e.g. accuracy) of registered information in the future. Such scenario will be possible by proposing and implementing new educational programs for people working in the health field. Suitable codes (e.g. orphan-codes and/or OMIM codes) must also be used for identifying and making rare diseases traceable. These improvements should facilitate exchanges across registries and facilitate government decisions leading to better health services for patients.

## Limitations

Although this study is the first initiative for systematically record rare diseases in Colombia under-reporting has been largely observed. This was mainly due to doctors’ limited experience and knowledge regarding rare diseases and by the fact that severe forms of numerous conditions naturally leads to poor outcomes and short-term survival, despite suitable management.

## Additional files

**Additional file 1: Figure S1.** Methodology for the collection of data included in the registry.

**Additional file 2.** Additional methods include two sections: establishing a national registry and statistical analysis.

## Abbreviations

RD-HUB: Rare Disease-HUB
NORD: National Organization for Rare Disorders
RDCRN: Rare Diseases Clinical Research Network
CHSPM: Colombian Health and Social Protection Ministry’s
SISPRO: Colombian integral social protection information system
SIVIGILA: Public Health Surveillance System
SD: standard deviation
ICD: International Statistical Classification of Diseases
