# Supplementary figures and images for: A first description of the Colombian national registry for rare diseases
Source: BMC Res Notes. 2017 Oct 26;10:514. doi: 10.1186/s13104-017-2840-1 (PMC5659024; doi:10.1186/s13104-017-2840-1)

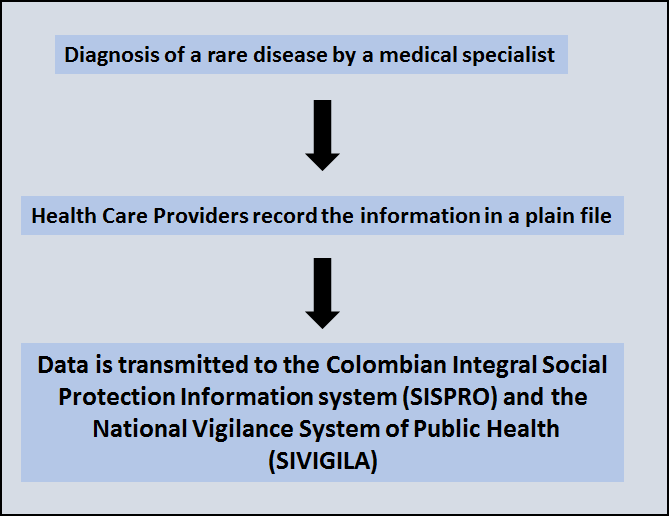

Supplement: Supplementary file 1 — Additional file 1: Figure S1. Methodology for the collection of data included in the registry. [file 13104_2017_2840_MOESM1_ESM.tif]
