# Supplementary material for: A first description of the Colombian national registry for rare diseases
Source: BMC Res Notes. 2017 Oct 26;10:514. doi: 10.1186/s13104-017-2840-1 (PMC5659024; doi:10.1186/s13104-017-2840-1)
Supplement: Supplementary file 2 — Additional file 2. Additional methods include two sections: establishing a national registry and statistical analysis. [file 13104_2017_2840_MOESM2_ESM.docx]

**Additional methods**

***Establishing a national registry***

Measures aimed to promote and improve health care of rare diseases were introduced in 2010 (law 1392) [1]. Rare diseases were categorized as representing *“special interest needing state protection”*. Law 1392 promoted the development of clinical guidelines for diagnosis, management, census and registry of patients suffering rare diseases. It also stated that the list of rare diseases was to be updated every two years. The complete list of rare diseases is available at the Colombian Health and Social Protection Ministry’s (CHSPM) webpage [2]. This registry attempts to create a basic information system about orphan diseases, affecting children and adults, providing a better insight regarding prevalence, mortality, geographic distribution in order to identify sanitary, social an scientific resources, as well as generating an integral and centered service for patients and their families.

A further law (law 1438, 2011) determined that rare diseases were severe, life-threatening and chronically debilitating conditions, having a prevalence of less than 1/5,000 (n=1,920 diseases) [3]. Colombia established a ten-year public health plan in 2013, which promoted the attention of distinct forms of disability and rare diseases. Statutory law 1751/2015 stated that people suffering from rare diseases require special protection by the state and that health care should not be limited by any type of administrative or financial restriction [4]. Following national laws on rare diseases, regional authorities created a technical committee concerning the bases for diagnosis, care, treatment and medical service availability.

Patient data obtained from clinical records at the time of enrolment in the national registry provided input for a cross-sectional study. Diseases were included according to the list previously described in resolution 430/2013 [2, 5]. The diagnosis was performed by medical specialists who included clinical details in the medical history. Then, patients´ data was sent to the Colombian Integral Social Protection Information system (SISPRO). In parallel, the National Vigilance System of Public Health (SIVIGILIA) was informed on the diagnosis new patients affected by rare diseases (**Additional file 1: Figure S1**). Extended information is available as a free internet resource [6-7]. The health care providers were responsible for recording patients´ information according to the clinical documentation stored in their files.

Data such as age, gender, disease, disabilities, type of health system affiliation, educational level, profession, ethnic origin, pharmacological treatment and geographic origin were thus registered.

Concerning personal data protection regulation, health insurers collected patients’ information regarding patients in line with law 1581/2012 which authorizes the use of personal information for statistical or scientific purposes [8]. Informed consent was not therefore mandatory. Data were handled and used exclusively for national registry purposes. Personal and sensitive data were stored in the CHSPM secure servers, protected by advanced firewall and technological systems. The initial census was incorporated into SISPRO which was used to establish the national registry for rare diseases (from a minimal data set). The CHSPM’s Epidemiology and Demography Direction and Information, Technology and Communication Office, collected the data according to previously standardized methods. Quality control procedures involved validating health insurer information as well as discovering potential duplications of patients´ identification. A second quality control was carried out by the CHSPM which involved crossing data in the registry with that available in the Colombian SISPRO database. Thirteen and 40 variables were registered concerning demographic and clinical condition variables, respectively. Data missing from the original report was standardized and uploaded to SISPRO. Private specialized companies performed the final quality control. The Institutional Ethics Committee of the National Institute of Health (INS) approved all of this study’s´ steps (Protocol: PRO-R02.55). For adults, as for participants younger than 16, the laws included in the Resolution 1954 (2012) were followed concerning informed consents. Therefore, informed consent was not mandatory for the creation of the registry.

***Statistical analysis***

Univariate descriptive statistics were used. Frequencies were used for describing categorical variables. Age was presented as the mean and standard deviation. R software v3.0.2 ([www.R-project.org](http://www.R-project.org)) was used for statistical analysis. The prevalence rates were calculated as the number of cases/population size. Information on the Colombian population size was extracted from the Colombian Department of Statistics (DANE).

**References**

1. https://www.minsalud.gov.co/sites/rid/Lists/BibliotecaDigital/RIDE/DE/DIJ/ley-1392-de-2010.pdf

12.<https://www.minsalud.gov.co/Normatividad_Nuevo/Resoluci%C3%B3n%202048%20de%202015.pdf>

13.<http://www.descentralizadrogas.gov.co//pdfs/politicas/nacionales/LEY_1438_DE_2011-Reforma_al_Sistema_General_de_Seguridad%20_Social_en_Salud.pdf>

14.https://www.minsalud.gov.co/Normatividad_Nuevo/Ley%201751%20de%202015.pdf

5. http://www.fecoer.org/download/Resoluci%C3%B3n%200430%20de%202013.pdf

6.<https://www.minsalud.gov.co/sites/rid/Lists/BibliotecaDigital/RIDE/DE/DIJ/Decreto-1954-de-2012.PDF>

7.[http://www.ins.gov.co/lineas-de-accion/Subdireccion Vigilancia/sivigila/Protocolos%20SIVIGILA/PRO%20Enfermedades%20hu%C3%A9 fanas%20y%20raras-.pdf](http://www.ins.gov.co/lineas-de-accion/Subdireccion%20Vigilancia/sivigila/Protocolos%20SIVIGILA/PRO%20Enfermedades%20hu%C3%A9%20fanas%20y%20raras-.pdf)

8. https://www.mintic.gov.co/portal/604/articles-4274_documento.pdf.
